# Reporters for the analysis of *N*-glycosylation in *Candida albicans*^☆^

**DOI:** 10.1016/j.fgb.2013.03.009

**Published:** 2013-07

**Authors:** Shahida Shahana, Hector M. Mora-Montes, Luis Castillo, Iryna Bohovych, Chirag C. Sheth, Frank C. Odds, Neil A.R. Gow, Alistair J.P. Brown

**Affiliations:** Aberdeen Fungal Group, School of Medical Sciences, University of Aberdeen, Institute of Medical Sciences, Foresterhill, Aberdeen AB25 2ZD, UK

**Keywords:** *Candida albicans*, Glycosylation, Cell wall, Glycosylation reporter

## Abstract

A large proportion of *Candida albicans* cell surface proteins are decorated post-translationally by glycosylation. Indeed *N-*glycosylation is critical for cell wall biogenesis in this major fungal pathogen and for its interactions with host cells. A detailed understanding of *N-*glycosylation will yield deeper insights into host-pathogen interactions. However, the analysis of *N-*glycosylation is extremely challenging because of the complexity and heterogeneity of these structures. Therefore, in an attempt to reduce this complexity and facilitate the analysis of *N-*glycosylation, we have developed new synthetic *C. albicans* reporters that carry a single *N-*linked glycosylation site derived from *Saccharomyces cerevisiae* Suc2. These glycosylation reporters, which carry *C.* *albicans* Hex1 or Sap2 signal sequences plus carboxy-terminal FLAG_3_ and His_6_ tags, were expressed in *C.* *albicans* from the *ACT1* promoter. The reporter proteins were successfully secreted and hyperglycosylated by *C.* *albicans* cells, and their outer chain glycosylation was dependent on Och1 and Pmr1, which are required for *N-*mannan synthesis, but not on Mnt1 and Mnt2 which are only required for *O-*mannosylation. These reporters are useful tools for the experimental dissection of *N-*glycosylation and other related processes in *C.* *albicans*, such as secretion.

## Highlights

• Reporters for dissection of *N-*glycosylation in *Candida albicans.*
• Detection of glycosylation at the single site on epitope-tagged reporter.
• Reporter faithfully reflects glycosylation defects in cell wall mutants.

## 1 Introduction

*Candida albicans* is an opportunistic pathogen that inhabits the oral cavity, gastrointestinal and urogenital tracts of many healthy individuals. This fungus is a frequent cause of mucosal infections (e.g. oral and vaginal thrush), and in severely immunocompromised patients it can cause life-threatening systemic infections of the bloodstream and internal organs (Calderon et al., 2003; Kabir and Hussain, 2009; Odds et al., 1988).

The *C.* *albicans* cell wall plays a key role in host-fungus interactions during the infection process. The *C.* *albicans* cell commits significant resources to the synthesis of its cell wall, which comprises approximately 30% of its dry weight. This robust cell wall helps to protect the fungus from environmental insults. Yet the cell wall is dynamic, its proteomic and carbohydrate content responding to the growth conditions (Lenardon et al., 2010; Sosinska et al., 2008). The cell wall is made up of an inner layer of chitin, ß-1,3-glucans and ß-1,6-glucans, and an outer layer that largely comprises highy glycosylated mannoproteins (Klis et al., 2001). These mannoproteins, which decorate the *C.* *albicans* cell surface, represent significant antigenic determinants that contribute to and modulate immune recognition (Mora-Montes et al., 2010, 2007; Netea et al., 2008; Torosantucci et al., 1990; Wang et al., 1998; Gow et al., 2012). Indeed, mannoproteins are important *C.* *albicans* pathogen-associated molecular patterns (PAMPs) that are recognised by specific host pattern recognition receptors (PRRs) during host innate immune responses (Brown and Gordon, 2001; Gow et al., 2007; McKenzie et al., 2010; Netea et al., 2008, 2006; Sheth et al., 2011; Gow et al., 2012). Cell surface mannoproteins also include adhesins that promote attachment to host tissues and invasins that enhance endocytosis by host cells (Bates et al., 2006, 2005; Mora-Montes et al., 2009; Munro et al., 2005; Phan et al., 2007; Sundstrom et al., 2002). Not surprisingly, *C. albicans* null mutants that lack key mannan biosynthetic enzymes display attenuated virulence, confirming that cell wall mannosylation is important for pathogenicity (Bates et al., 2006, 2005; Mora-Montes et al., 2010, 2007, 2009).

*C. albicans* cell surface mannoproteins are posttranslationally adorned with *O-* and/or *N-*mannans that influence their biophysical properties, stability and function (Harvey, 2005). In addition, ß-1,2-linked mannosides are attached to phosphopeptidomannan and phospholipomannan molecules in the *C. albicans* cell wall (Fradin et al., 2008). Here we focus on *N-*glycosylation, which has been studied in some depth in *C. albicans* (Bates et al., 2006, 2005; Mora-Montes et al., 2010, 2007; Nishikawa et al., 2002; Southard et al., 1999; Warit et al., 2000). In general, the process of *N-*glycosylation in *C. albicans* appears to be similar to that of the non-pathogenic yeast, *Saccharomyces cerevisiae* although there are some important differences in the *N-*mannans in these two species. *N-*glycosylation initiates in the endoplasmic reticulum where a preassembled *N-*linked glycan core (Glc_3_Man_9_GlcNAc_2_) is transferred onto Asn*-*X-Ser/Thr acceptor sites (Burda et al., 1999; Knauer and Lehle, 1999). Glucosidases and mannosidases then process this inner core glycan to Man_8_GlcNAc_2_ (Herscovics, 1999) before transit to the Golgi apparatus where elaborated outer chains are added. Linear backbones of up to and over 50 α-1,6-linked mannose residues are laid down on the inner core. Highly branched structures are then created by addition of α-1,2- and α-1,3-linked mannoses (Ballou, 1990; Herscovics, 1999; Herscovics and Orlean, 1993). In *C. albicans*, outer chains also contain mannosylphosphate, which are chains of ß-1,2-linked mannoses attached via phosphodiester bonds (Trinel et al., 1997). Together, these carbohydrate decorations can comprise up to 95% of the molecular mass of cell wall mannoproteins (Dean, 1999).

*N-*glycosylation is dependent upon a number of activities, some of which have been defined in *C. albicans*. The generation of outer chains is dependent on Och1, which adds the first α-1,6-linked mannose to the inner core. Therefore inactivation of Och1 blocks the elongation of *N-*mannans in *C. albicans* (Bates et al., 2006). Members of several families of glycosyl transferases, including *MNN*, *BMT* and *MNT* genes, mediate the addition of α-1,2-, ß-1,2-, α-1,3- and α-1,6-linked mannose residues during *N-* and *O-*glycosylation. Most of them remain to be characterised (Mora-Montes et al., 2010). However, Mnt1 and Mnt2 have been defined as functionally redundant α-1,2-mannosyltransferases that add the second and third mannose units during *O-*glycosylation (Buurman et al., 1998; Munro et al., 2005; Thomson et al., 2000). Inactivation of *MNT1* and/or *MNT2* does not appear to affect *N-*glycosylation. Mannosyltransferases depend upon Mn^++^ for their activity, and hence upon the Golgi Mn^++^ transporter, Pmr1. Hence *C. albicans pmr1Δ* mutants display defects in *N-*linked outer chain glycosylation as well as in *O-*glycosylation (Bates et al., 2005).

As these and other components of the glycosylation apparatus are defined, the next step involves the detailed biochemical dissection of their contribution to *N-*glycosylation and to cell surface properties. This is not trivial, however, because glycosylation patterns are complex and heterogeneous, as described for invertase in *S. cerevisiae*, for example (Zeng and Biemann, 1999). Different cell wall proteins carry different numbers of potential *N-*glycosylation sites. Even specific cell wall proteins display highly heterogeneous glycosylation patterns (Harvey, 2005; Medzihradszky, 2008), potentially because of the differential usage of potential *N-*glycosylation sites on individual molecules. The situation may be complicated further by possible stochastic behaviours of the mannosylation apparatus leading to the elaboration of outer chains of variable lengths and branching patterns. Clearly, specialised tools are required to reduce this complexity thereby allowing biochemical dissection of *N-*mannans. Therefore we now describe the development and validation of a new reporter for the analysis of *N-*glycosylation in *C. albicans*.

## 2 Materials and methods

### 2.1 Strains and growth conditions

Strains used in this study are listed in Table 1. Strains were grown at 30 °C in YPD or SD minimal medium (Sherman, 1991) supplemented with 50 μg/ml uridine as required.

### 2.2 Strain construction

Three glycosylation reporter genes (GR1, GR2 and GR3) were designed, synthesised by DNA2.0 (Menlo Park, CA, USA) and subcloned between the HindIII and NheI sites of pACT-GFP (Barelle et al., 2004), which is based on CIp10. CIp10 is a *Candida* integrating plasmid that was developed for the stable integration of sequences into the *C. albicans* genome at the *RPS1* locus (formally known as *RPS10*) (Murad et al., 2000). The GR sequences replaced the GFP coding region in pACT-GFP. This placed each reporter gene under the control of the *C. albicans ACT1* promoter and *S. cerevisiae CYC1* terminator in the plasmids pACT-GR1, pACT-GR2 and pACT-GR3, respectively. Each gene was resequenced to confirm the accuracy of the gene construction. The sequences of the GR1, GR2 and GR3 reporter genes are available with GenBank Accession Nos. GU733317, GU733318, and GU733319, respectively. GR protein structure was predicted using the UCSF Chimera modeller program (Yang et al., 2012).

Each plasmid was linearised by digestion with StuI and transformed into *C. albicans* (Gietz et al., 1995; Walther and Wendland, 2003) to target chromosomal integration of the constructs at *RPS1* (Murad et al., 2000). Correct integration at *RPS1*, which was confirmed by diagnostic PCR, restores *URA3* functionality and does not impair *in vitro* or *in vivo* phenotypes (Brand et al., 2004).

### 2.3 Protein preparations

Intracellular protein extracts were prepared using conventional protocols (Millar et al., 1992). Briefly, *C. albicans* cells were grown in YPD overnight, harvested, resuspended in lysis buffer (0.1 M Tris–HCl, pH 8, 10% glycerol, 1 mM DTT, 0.1 mg/ml pepstatin A, containing protease inhibitor cocktail (Roche Applied Science; Burgess Hill, UK)), and sheared with glass beads. Lysates were centrifuged at 15,000*g* for 10 min at 4 °C, and these extracts stored at −20 °C. Extracts were reduced with 3 mM dithiothreitol (60 °C, 20 min), S-alkylated with 13 mM iodoacetamide (25 °C, 10 min), digested 8 h at 37 °C with trypsin (20 ng/μl; Promega, UK), dried by rotary evaporation (SC110 Speed Vac, USA), and dissolved in 0.1% formic acid before analysis by western blotting.

To isolate extracellular proteins, *C. albicans* strains were grown in 1 l SD at 30 °C for 24 h. The culture medium was separated from cells by centrifugation, the extracellular proteins concentrated using an Amicon membrane ultrafiltration system (10 kDa cut-off: Millipore Ltd, Watford, UK) as described (Schratter, 2004), and the extracellular fraction stored at −20 °C. To purify the His_6_-tagged reporter proteins, His60 Ni Gravity Column Purification kits were used according to the manufacturer’s instructions (Takara Bio Europe/Clontech, Saint-Germain-en-Laye, France). Preparations were then dialysed against phosphate-buffered saline and freeze-dried before analysis or concentrated by Vispaspin ultrafiltration (Sartorius Stedim Biotechnology, UK).

### 2.4 Western blotting

Protein concentrations were assayed using standard protocols (Bradford, 1976). In some cases, before electrophoresis, samples were treated with 25 mU of endoglycosidase H (Roche) in 50 mM sodium acetate buffer, pH 5.2, for 16 h at 37 °C to remove *N-*mannan. Standard protocols were used for western blotting (Smith et al., 2004) with slight modifications. For extracellular fractions, samples corresponding to equivalent culture volumes were subjected to electrophoresis. Protein samples were mixed with NuPAGE® sample loading buffer (Life Technologies Ltd., Paisley, UK) containing 1 mM DTT and heated at 70 °C for 10 min. Proteins were separated on 4–12% NuPAGE Bis-Tris gels (Invitrogen) for 1 h at 200 V/cm, and electro-transferred to polyvinylidene difluoride (PVD) membranes for 3 h at 25 V/cm. Parallel gels were stained with Coomassie reagent or with the Pierce Glycoprotein staining kit (Fisher Scientific, Loughborough, UK). PVD membranes were blocked overnight at 4 °C using PBS plus 0.1% Tween 20 and 5% semi-skimmed dry milk. Western blots were either probed with a rabbit anti-FLAG polyclonal antibody (1:10,000 dilution in PBS; Sigma–Aldrich, Gillingham, UK), or a horse radish peroxidise-conjugated mouse anti-His_6_ antibody (1:5000 dilution in PBS; Invitrogen). The secondary antibody was horse radish peroxidise-conjugated anti-rabbit IgG (1:3000 dilution in PBS; Cell Signalling, MA, USA). Signals were detected with an HRP western blotting kit (Amersham, Little Chalfont, Buckinghamshire, UK).

## 3 Results

### 3.1 Design of synthetic, codon*-*optimised *N-*glycosylation reporters for *C. albicans*

**Fig. 1:**
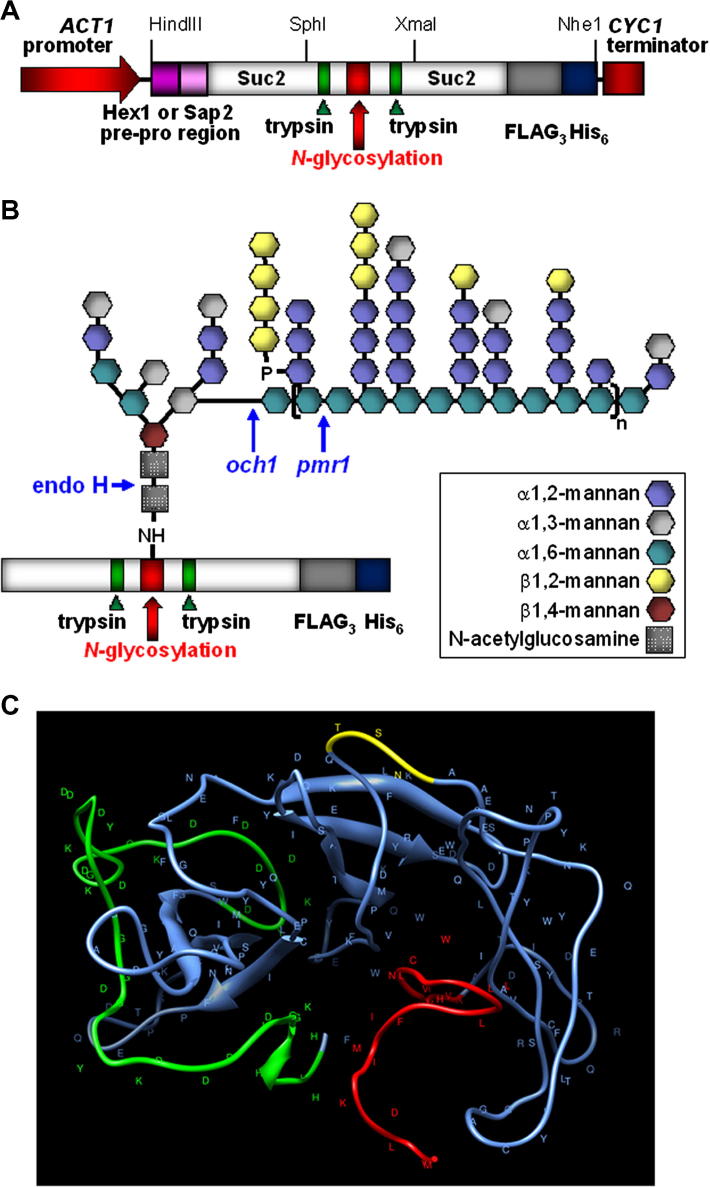
Design of the *N*-glycosylation reporters for *C. albicans*. (A) Cartoon illustrating the structure of the gene, which is transcribed from the *ACT1* promoter and terminated via Sc*CYC1* sequences in pACT1 (Barelle et al., 2004). The codon optimised, synthetic coding regions encode part of the ScSuc2 protein which includes a single *N*-glycosylation site (Asn146-Ser-Thr) (Ziegler et al., 1988) flanked by trypsin cleavage sites. The synthetic coding regions encode carboxy-terminal FLAG3 and His6 tags. The GR1 reporter gene encodes an amino-terminal signal sequence from Hex1 (Cannon et al., 1994), whilst GR2 encodes an amino-terminal pre-pro-sequence from Sap2 (Morrison et al., 1993; Togni et al., 1991). GR3 is derived from GR2, but lacks the *N*-glycosylation site. (B) Cartoon illustrating the predicted structure of a mature *N*-glycosylated reporter protein, showing the glycosylation defects in *C. albicans och1* and *pmr1* mutants and the site of cleavage of endoglycosidase H (Netea et al., 2008). (C) Structural prediction of the GR1 reporter protein, highlighting the glycosylation site (yellow), the signal sequence (red), and the FLAG3-His6 tag (green).

Our goal was to develop a new reporter to facilitate the analysis of *N-*glycosylation in *C. albicans*. We reasoned that this reporter need not encode a functional entity, except with regard to its ability to act as a glycosylation target. Therefore, we chose to create a synthetic reporter that carries multiple features to facilitate downstream characterisation of its *N-*glycosylation. This reporter carries a single *N-*glycosylation site (to prevent heterogeneous glycosylation at multiple target sites). This site, which is derived from *S. cerevisiae* Suc2, has been validated biochemically (Marshall, 1972; Gavel and von Heijne, 1990). We chose a 147 amino acid region of *S. cerevisiae* Suc2 that surrounds a well-defined glycosylation site (Asn146-Ser-Thr) in the Suc2 protein (Ziegler et al., 1988). This Suc2 fragment, which corresponds to amino acids 119 to 265 of the unprocessed invertase sequence, carries trypsin cleavage sites seven residues before (K139) and five residues after (R151) the target *N-*glycosylation site to facilitate downstream analysis by mass spectroscopy. The next step was to include a signal sequence to programme secretion of the reporter protein. The first reporter we constructed (GR1) included the 23 amino*-*terminal amino acids of *C. albicans N-*acetylglucosaminidase Hex1 (Cannon et al., 1994). Finally, to facilitate immunodetection and purification, three sequential eight amino acid FLAG epitopes and a single His_6_ sequence were included at the carboxy terminus of this synthetic reporter, each of which were separated by a glycine residue (Fig. 1).

This artificial amino acid sequence was then converted into its corresponding nucleotide open reading frame, using preferred codons for *C. albicans* (Brown et al., 1991) and avoiding usage of the CTG codon, which is decoded as serine rather than leucine in this pathogen (Santos et al., 1993; White et al., 1995). SphI and XmaI restriction sites were then introduced into the GR1 sequence upstream and downstream of the region encoding the target *N-*glycosylation site to facilitate modification of this site in the future. Also HindIII and NheI sites were introduced at the beginning and end of the synthetic coding region to facilitate cloning (Fig. 1). Unwanted restriction sites were then removed by exchanging synonymous codons. Having designed this artificial reporter, the GR1 gene was then synthesized and cloned into the *C. albicans* expression vector pACT1-GFP (Barelle et al., 2004) to create pACT1-GR1, by replacing GFP with the GR1 reporter downsteam of the *ACT1* promoter (Section 2).

Two additional reporters (GR2 and GR3) were then designed, synthesized and cloned to generate the plasmids pACT1-GR2 and pACT1-GR3. In GR2 the 23 residue Hex1 signal sequence was replaced with the 63 amino acid amino*-*terminal region from the *C.* *albicans* Sap2 protein that contains the pre-pro*-*peptide sequence from this secreted aspartyl protease (Morrison et al., 1993; Togni et al., 1991) (Fig. 1). GR3 was based on GR2, but in GR3 the glycosylation target Asn146-Ser-Thr was changed to Gly146-Ser-Thr. GR3 is therefore a negative control which contains Sap2 secretion signals but lacks the target *N-*glycosylation site.

### 3.2 Expression of the *N-*glycosylation reporters in *C. albicans*

On the basis of their design, the GR1, GR2 and GR3 reporters were expected to express immature (unprocessed) proteins in *C. albicans* of about 25 kDa, 28 kDa and 28 kDa, respectively (Fig. 1). Following signal peptide cleavage the GR1, GR2 and GR3 proteins were predicted to have molecular masses of about 23 kDa, 26 kDa and 26 kDa, and following cleavage of the Sap2 pro*-*peptides from GR2 and GR3, these proteins were expected to have masses of about 23 kDa in the absence of any glycosylation. GR1 and GR2 contain the target Asn146-Ser-Thr sequence and therefore were predicted to be *N-*glycosylated, whereas GR3 lacks this sequence and was expected to remain unglycosylated.

**Fig. 2:**
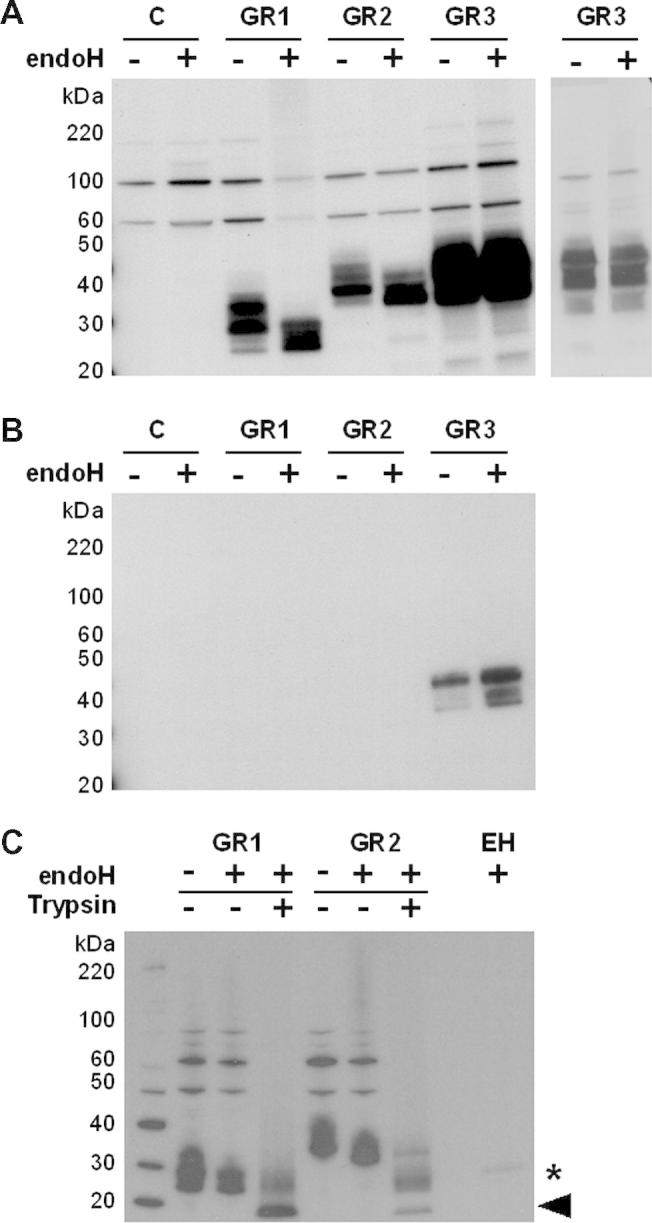
Intracellular forms of the *N*-glycosylation reporters in *C. albicans*. Western blots of intracellular extracts of wild type *C. albicans* cells (CAI4) transformed with pACT-GR1, pACT-GR2, pACT-GR3 or the empty CIp10 vector (C, control) (Table 1). Extracts were either untreated (−) or treated (+) with endoglycosidase H before analysis. (A) Western blots were probed with a polyclonal anti-FLAG antibody. In the left panel, 15 μg of protein sample was loaded per lane. The right panel shows a separate gel containing less protein (5 μg) for the GR3 protein extracts. (B) An analogous western blot probed with a monoclonal anti-His6 antibody. (C) Western blot of trypsin digested intracellular extracts from wild type *C. albicans* cells (CAI4) expressing pACT-GR1 or pACT-GR2 and probed with anti-FLAG antibody. The carboxy-terminal FLAG-tagged GR tryptic peptides of about 15 kDa are highlighted (arrow) as well as the endoglycosidase H band at about 29 kDa (asterisk). The Coomassie stained gel confirming the protein loading is shown in the supplementary data (Fig. S1).

**Fig. 3:**
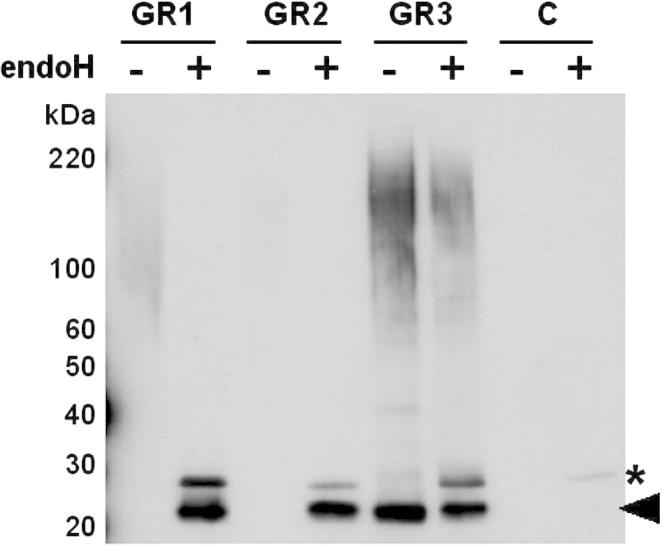
Secreted forms of the *N*-glycosylation reporters in *C. albicans*. Western analysis of secreted proteins from wild type *C. albicans* cells (CAI4) transformed with pACT-GR1, pACT-GR2, pACT-GR3 or the empty CIp10 vector (C, control) probed with the polyclonal anti-FLAG antibody. Extracts were either treated (+) or not treated (−) with endoglycosidase H before analysis and samples corresponding to equivalent culture volumes were run on the gels. The black arrow indicates the mature 23 kDa deglycosylated forms of the GR proteins. The asterisk highlights the band corresponding to endoglycosidase H itself.

To test these predictions we first examined the intracellular expression of the GR1, GR2 and GR3 reporters. Total soluble protein extracts were prepared from *C.* *albicans* CAI4 cells transformed with pACT1-GR1, pACT1-GR2 or pACT1-GR3, and from control cells lacking GR sequences (Section 2). Western blotting was then performed on these extracts, probing for the FLAG_3_ and His_6_ epitopes (Fig. 2A and B). Using the anti-FLAG antibody, clear expression of GR proteins was observed in pACT1-GR1, pACT1-GR2 and pACT1-GR3 transformants compared with the control cells. The intracellular levels of GR3 were higher than for GR1 and GR2. GR1 proteins of about 25–38 kDa were observed, compared with GR2 proteins of about 32–40 kDa and GR3 proteins of about 22–40 kDa. The main intracellular forms observed for GR3 (30–40 kDa) were longer than predicted (26–28 kDa) (Fig. 2A). This might reflect unexpected effects on the electrophoretic mobility of intracellular GR3 because these bands were also detected with the anti-His_6_ antibody (Fig. 2B), their mobility was not affected by treatment with endoglycosidase H (Fig. 2A and B), and the mobility of the secreted form of GR3 displayed the expected mass of about 23 kDa (Fig. 3). We conclude that all three GR reporters were successfully expressed in *C. albicans*.

Treatment of these extracts with endoglycosidase H (Mora-Montes et al., 2007) (which removes asparagine-linked mannosylation) reduced the masses of the GR1 and GR2 proteins, but not the GR3 proteins (Fig. 2A). Therefore, we conclude that GR1 and GR2 were *N-*glycosylated, and that GR3 was not glycosylated, as predicted. Some heterogeneity in the lengths of the GR proteins remained after deglycosylation with endoglycosidase H (Fig. 2A). This suggests that both processed and unprocessed forms of each GR protein (retaining or lacking the amino*-*terminal signal peptide) were present inside *C. albicans* cells. Proteolysis is unlikely to account for the multiple isoforms as protease inhibitors were included during protein extraction (Section 2). Interestingly, the full length form of GR3 (about 40 kDa) was present at significantly higher levels than the processed forms of GR3. In contrast the processed forms of GR2 (about 32 kDa) were more abundant than the unprocessed forms of this protein. Therefore signal peptide cleavage from the GR2 reporter appeared more efficient than for the GR3 protein.

The anti-His_6_ antibody appeared less sensitive than the anti-FLAG antibody at least with regard to detection of the GR proteins as signals were detected for GR3, but not for GR1 and GR2 (Fig. 2B). Therefore anti-FLAG antibody was used for most of our subsequent experiments. Nevertheless, these analyses confirmed the lack of glycosylation of GR3 and the observation that the unprocessed form of GR3 accumulates inside *C. albicans* cells.

The functionality of the tryptic cleavage sites (Fig. 1A) was tested by digestion of GR1 and GR2. As predicted, carboxy-terminal FLAG_3_-tagged fragments of about 15 kDa were generated by tryptic digestion of these reporter proteins (Fig. 2C). Coomassie staining of a parallel gel confirmed the efficacy of the tryptic digestion on the proteins in these extracts as well as the comparable protein loading of GR1 and GR2 extracts (Fig. S1).

### 3.3 Secretion of the *N-*glycosylation reporters from *C.* *albicans*

Having confirmed that all three GR reporters are expressed in *C.* *albicans*, that GR1 and GR2 are glycosylated at the target Asn146, and that GR3 is a valid negative control for glycosylation, the next step was to examine the secreted forms of these reporters. Secreted proteins were harvested from the growth medium of *C.* *albicans* cells transformed with pACT1-GR1, pACT1-GR2 or pACT1-GR3, and from control cells that did not contain a GR reporter. These secreted extracts were then subjected to western blotting, probing for the FLAG epitope (Fig. 3). A heterogeneous smear of high molecular weight material (>60 kDa) was reproducibly detected in GR1 extracts, and an analogous, faint smear was barely detectable in GR2 extracts. These probably represent hyperglycosylated forms of the GR1 and GR2 proteins. Consistent with this conclusion, endoglycosidase H treatment of these GR1 and GR2 extracts resolved this heterogeneous material into clear bands of about 23 kDa. These 23 kDa bands were consistent with the generation of processed, deglycosylated GR1 and GR2 proteins of the predicted molecular mass. A second band of 25 kDa was observed following endoglycosidase H treatment. This band was also observed in control extracts that lack GR sequences (Fig. 3), and when running endoglycosidase H alone. We conclude that this background band represents non*-*specific antibody binding to endoglycosidase H itself (Umeyama et al., 2002).

The GR3 intracellular extracts also displayed a heterogeneous smear of high molecular weight material (>35 kDa: Fig. 3). However, this smear was not resolved following endoglycosidase H treatment, and the nature of this material is not known. Instead, a GR3-dependent band of about 23 kDa was observed in extracellular fractions irrespective of whether they were treated with endoglycosidase H or not (Fig. 3). We conclude that *C.* *albicans* cells can process and secrete some non*-N-*glycosylated GR3 protein. Therefore, all three GR reporters were secreted by *C.* *albicans*, GR1 and GR2 generating hyperglycosylated forms, as predicted.

### 3.4 Impact of specific glycosylation defects upon the *N-*glycosylation of GR1 in *C.* *albicans*

To further validate the GR reporters we tested the impact of well-defined glycosylation mutations upon their behaviour in *C.* *albicans*. First we focused on GR1, which carries the Hex1 signal sequence.

**Fig. 4:**
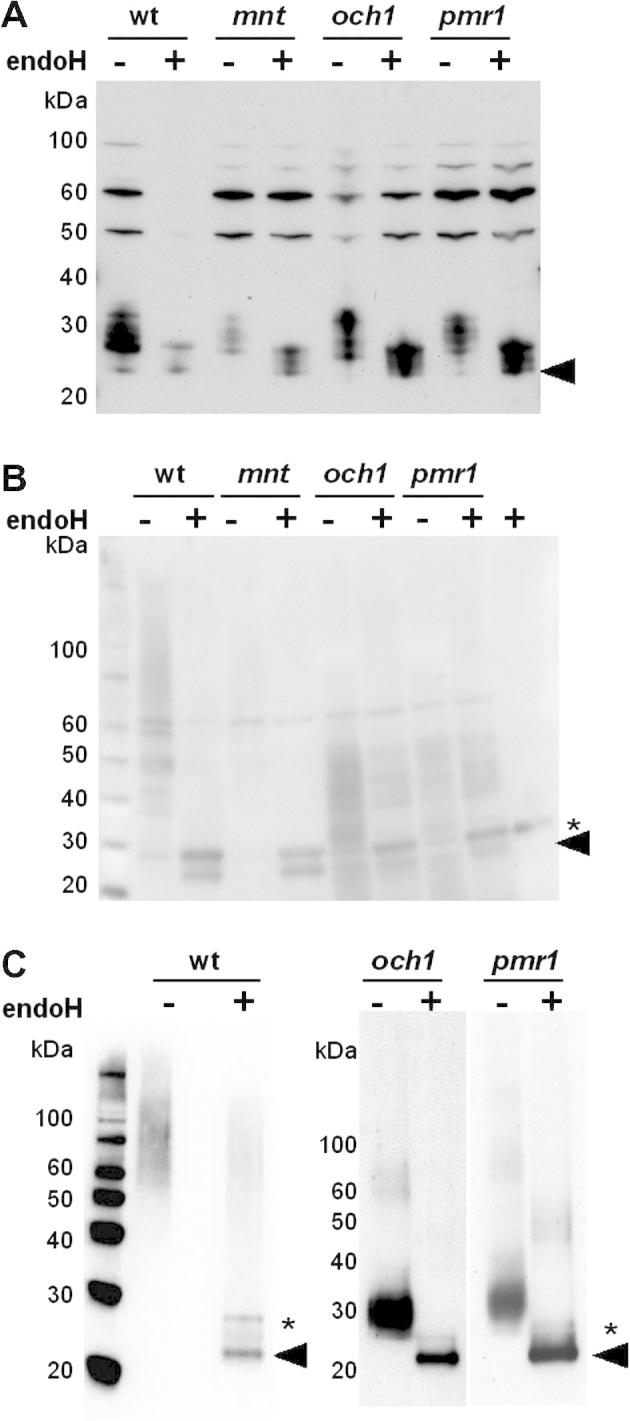
Impact of glycosylation defects upon GR1 processing in *C. albicans*. Western analyses of endoglycosidase H treated (+) and untreated (−) extracts from *C. albicans* mutants transformed with pACT1-GR1: wt, CAI4 cells transformed with pACT1-GR1; *mnt*, NGY112 cells (*mnt1 mnt2*) containing pACT1-GR1; *och1*, NGY205 cells containing pACT1-GR1; *pmr1*, NGY98 cells containing pACT1-GR1 (Table 1). Membranes were probed with the polyclonal anti-FLAG antibody. (A) Western analysis of intracellular protein extracts. (B) Western analysis of extracellular fraction. Samples corresponding to equivalent culture volumes were run on these gels. (C) Western analyses of His6-purified material from extracellular fractions from wild type, *och1* and *pmr1* cells: −, untreated; +, digested with endoglycosidase H. The black arrows highlight the processed, deglycosylated 23 kDa form of GR1. The asterisks highlight the background band corresponding to endoglycosidase H.

As described above, *C.* *albicans* cells lacking the functionally redundant *α*1,2-mannosyltransferases Mnt1 and Mnt2 display defects in *O-*glycosylation, but not *N-*glycosylation (Buurman et al., 1998; Munro et al., 2005; Thomson et al., 2000). Therefore, similar GR1 glycosylation patterns were predicted in wild type and *mnt1 mnt2* cells. Similar intracellular forms of GR1 were observed in both cell types, and these forms were resolved by endoglycosidase H into the 25 kDa protein retaining the signal sequence and the mature 23 kDa protein (Fig. 4A). Furthermore, the heterogeneous high molecular mass extracellular forms of GR1 secreted by wild type and *mnt1 mnt2* cells were resolved into the 23 kDa mature protein by treatment with endoglycosidase H (Fig. 4B). We conclude that, as predicted, the disruption of *MNT1* and *MNT2* did not affect GR1 glycosylation.

Both Och1 and Pmr1 are required for the elongation of *N-*mannan outer chains (Bates et al., 2006, 2005). However, neither *Och1* nor *Pmr1* is required for the addition of the inner core. Once again, the GR1 reporter behaved as predicted. No significant differences in the intracellular forms of GR1 were observed between wild type, *och1* and *pmr1* cells (Fig. 4A), suggesting that addition of the inner core glycosyl unit to GR1 remained unaffected by inactivation of Och1 or Pmr1. The extracellular extracts from *och1* and *pmr1* cells contained heterogeneous immunoreactive material of 30–50 kDa (Fig. 4B). Some of this secreted material appeared to be resistant to deglycosylation by endoglycosidase H, suggesting that it represented background material of some sort. However endoglycosidase H treatment did generate a FLAG-reactive band of about 23 kDa, presumably corresponding to the mature deglycosylated GR1 protein (Fig. 4B). To clarify this further, His_6_-tagged proteins were partially purified from the extracellular fractions of wild type, *och1* and *pmr1* cells (Section 2) and subjected to western blotting with the anti-FLAG antibody (Fig. 4C). Heavily glycosylated forms of the FLAG_3_-His_6_-tagged GR1 of greater than 55 kDa were secreted from wild type cells, whereas the major glycosylated forms of the GR1 protein that were secreted from *och1* and *pmr1* cells displayed molecular masses of about 30 kDa. All of these forms were resolved to the mature 23 kDa protein upon endoglycosidase H treatment. This indicates that, as predicted, the inactivation of Och1 or Pmr1 inhibited the elaboration of outer chain *N-*mannan on GR1.

### 3.5 Impact of glycosylation mutants upon GR2 *N-*glycosylation in *C. albicans*

**Fig. 5:**
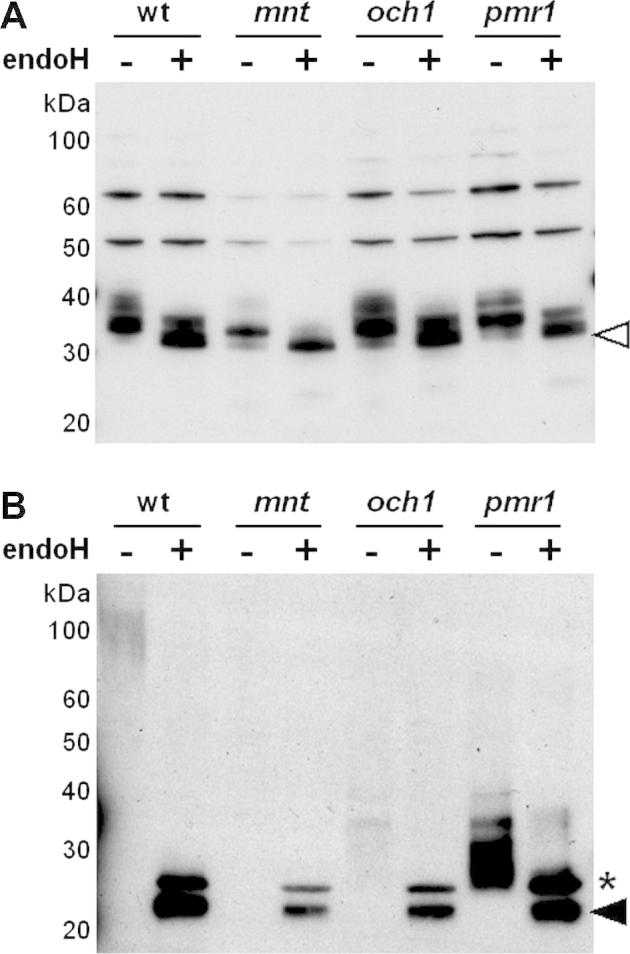
Effects of aberrant glycosylation upon GR2 processing in *C. albicans*. Western analyses of endoglycosidase H treated (+) and untreated (−) extracts from *C. albicans* mutants transformed with pACT1-GR2: wt, CAI4 cells transformed with pACT1-GR2; *mnt*, NGY112 (*mnt1 mnt2*) containing pACT1-GR2; *och1*, NGY205 containing pACT1-GR2; *pmr1*, NGY98 containing pACT1-GR2 (Table 1). Membranes were probed with the polyclonal anti-FLAG antibody. (A) Western analysis of intracellular protein extracts. (B) Western analysis of extracellular fraction. Samples corresponding to equivalent culture volumes: black arrow, the processed, deglycosylated 23 kDa form of GR2; white arrow, the unprocessed, deglycosylated 28 kDa pre-pro-form of GR2; asterisk, background band corresponding to endoglycosidase H.

Next we examined GR2 glycosylation in the *C. albicans och1*, *pmr1* and *mnt1 mnt2* mutants. Once again, the inactivation of Och1, Pmr1 or Mnt1 plus Mnt2 did not significantly affect the intracellular glycosylation patterns of GR2 (Fig. 5A), confirming that inner core glycosylation of GR2 proceeded normally in these mutants. However, effects upon the addition of mannan outer chains were observed when the secreted forms of GR2 were examined (Fig. 5B). Wild type and *mnt1 mnt2* cells displayed similar glycosylation patterns for the GR2 reporter. These cells secreted heterogeneous, hyperglycosylated, high molecular weight forms of GR2, which appear to be resolved to a fully processed 23 kDa form (Fig. 5B). However, we are unable to exclude the possibility that unprocessed forms of GR2 contribute to the upper band ascribed to endoglycosidase H. Lower yields of the hyperglycosylated reporter were generally obtained from the *mnt1 mnt2* double mutant. The basis for this is not known, but it is conceivable that preventing normal *O-*glycosylation might disturb indirectly the accumulation of *N-*glycosylated proteins. However, the processing of GR1 and GR2 did not seem to be grossly affected (Figs. 4B and 5B). In contrast, both *och1* and *pmr1* cells secreted a heterogeneous mixture of ca. 30–40 kDa forms of GR2 which, when treated with endoglycosidase H, were resolved to mature 23 kDa. The yields of untreated glycosylated GR2 were higher from the *pmr1* cells (Fig. 5B). Also, changes in mass resulting from glycosylation may not be reflected accurately in corresponding mobility changes on SDS gels. Nevertheless, these observations suggest that, like the GR1 reporter, the outer chain glycosylation of GR2 is inhibited by inactivation of Och1 or Pmr1.

## 4 Discussion

The *N-*glycosylation of cell wall and secreted proteins plays major roles in maintaining the physiological robustness of *C. albicans* cells and their interactions with host cells during disease progression (Bates et al., 2006, 2005; Mora-Montes et al., 2010, 2007; Munro et al., 2005; Phan et al., 2007; Sundstrom et al., 2002). Indeed, cell surface *N-*glycosylation is essential for pathogenicity (Bates et al., 2006, 2005; Mora-Montes et al., 2007, 2009; Munro et al., 2005) and contributes significantly to the recognition of *C. albicans* cells by host immunological defences (Mora-Montes et al., 2010, 2007; Netea et al., 2008; Torosantucci et al., 1990; Wang et al., 1998). A complete understanding of the impact of specific *N-*glycosylation events upon cell wall architecture, adhesion, and the endocytosis and immune recognition by host cells will depend upon detailed biochemical dissection of these *N-*glycosylation events. However, this is extremely challenging, largely because of the great complexity of *N-*glycosylation patterns at the *C. albicans* cell surface. Therefore, in this study we have developed and validated reporters designed to partially reduce this complexity and facilitate the analysis of *N-*glycosylation in *C. albicans*.

The synthetic GR1 and GR2 reporters were designed to carry a single *N-*glycosylation site to minimise the differential decoration of multiple potential sites within the target protein. This Asn146-Ser-Thr site was based on a known *N-*glycosylation site from *S. cerevisiae* Suc2 (Ziegler et al., 1988). To validate the GR1 and GR2 reporters we first examined their glycosylation patterns in wild type cells. We showed that both reporters are expressed in *C. albicans*, that partially processed forms of these proteins carrying an *N-*linked glycan core accumulate inside the cell, and that heterogeneous, hyperglycosylated GR1 and GR2 proteins are secreted successfully by *C. albicans* cells (Figs. 2 and 3). The GR1 and GR2 reporters were expressed and secreted with differing efficiencies as revealed by our western blotting of samples corresponding to equivalent culture volumes from each *C. albicans* strain.

To confirm that the Asn146-Ser-Thr site represented the only site of *N-*glycosylation within the GR1 and GR2 reporters, we compared their glycosylation patterns with those of a negative control. The GR3 reporter was identical to the GR2 reporter, except that the Asn146-Ser-Thr sequence was changed to Gly146-Ser-Thr. The secretion of mature, unglycosylated GR3 (23 kDa) by *C. albicans* cells (Fig. 3) confirmed that GR3 was not glycosylated, and hence that GR1 and GR2 were primarily hyperglycosylated at Asn146. We then further validated the GR1 and GR2 reporters by examining their glycosylation in well-defined glycosylation mutants (Figs. 4 and 5). Although their yields were slightly reduced, as predicted the glycosylation of GR1 and GR2 were not significantly affected in *C. albicans mnt1 mnt2* cells, in which *O-*glycosylation is disrupted (Buurman et al., 1998; Munro et al., 2005; Thomson et al., 2000).

**Fig. 6:**
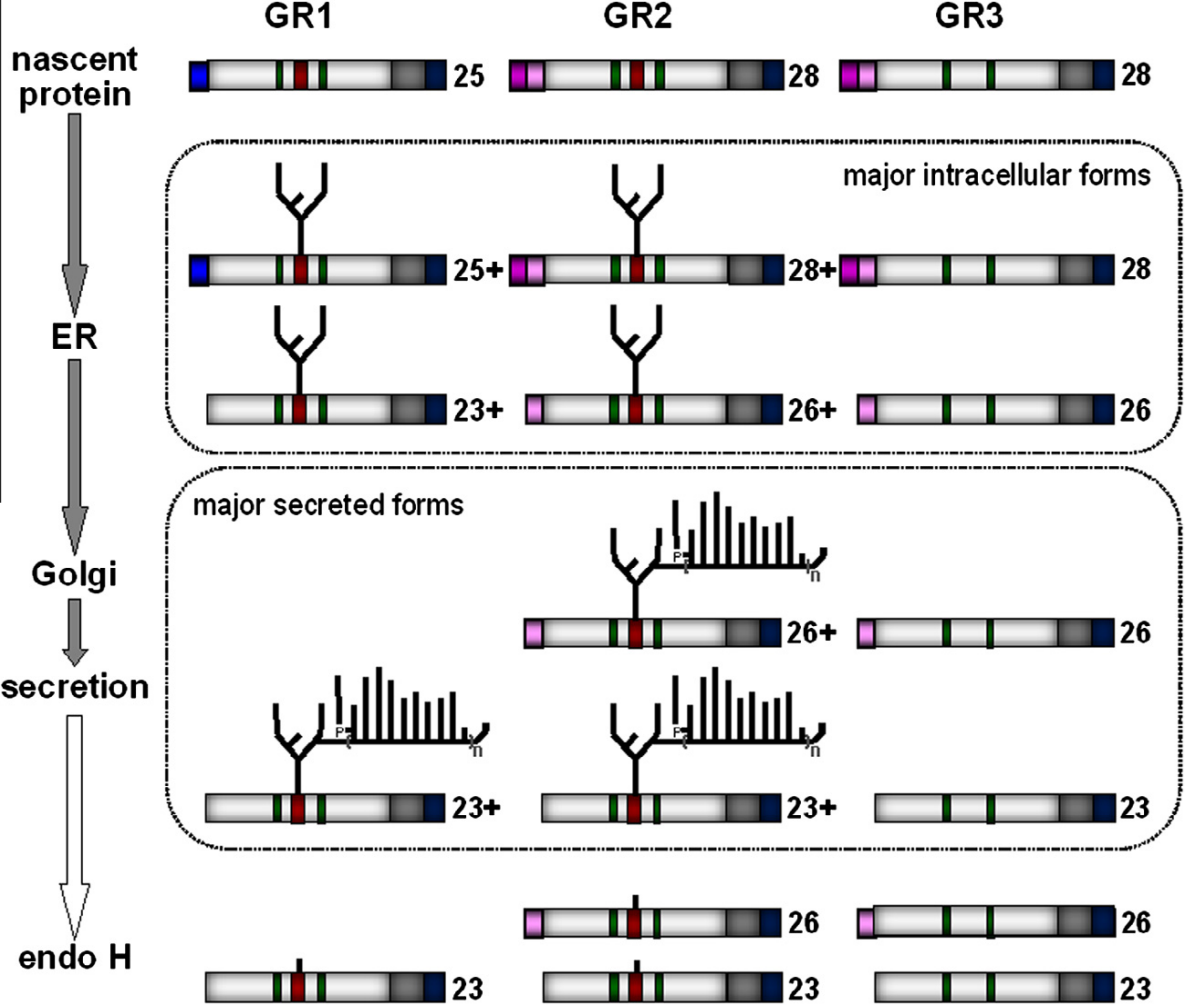
Cartoon summarising the observed forms of the GR1, GR2 and GR3 reporters in *C. albicans*. Each bar represents the structure of a GR protein. (Refer to Fig. 1 for the elements in each protein.) The number to the right of each bar indicates the estimated molecular mass, where + indicates apparent increased mass due to glycosylation. The nascent GR1 and GR2 proteins undergo core glycosylation, presumably in the ER, and a proportion of these proteins have their signal sequences removed (Fig. 2). Note that GR3 lacks the consensus *N*-glycosylation site and is not glycosylated (Fig. 2). The elaboration of the outer chains on GR1 and GR2 occurs, presumably in the Golgi apparatus, in an Och1 and Pmr1 dependent manner (Figs. 4 and 5). Also, the Sap2 pro-region is cleaved from a proportion of the GR2 and GR3 proteins. These various forms of GR protein are then secreted (Figs. 3–5). Subsequent endoglycosydase H treatment deglycosylates these GR proteins (Figs. 3, 4B and 5B). The major forms of each GR protein observed in the intracellular and extracellular fractions are highlighted by the boxes.

However, the elaboration of outer mannan chains on GR1 and GR2 was compromised in *C. albicans och1* and *pmr1* cells, which have been shown to display significant defects in *N-*mannan outer chain elongation (Bates et al., 2006, 2005). Therefore, the processing of the GR1 and GR2 proteins appears to reflect that of natural mannoproteins (Fig. 6) suggesting that they will prove useful reporters of *N-*glycosylation in *C. albicans*. The GR1 and GR2 reporters only differ with respect to their amino*-*terminal sequences. GR1 carries the Hex1 signal sequence whereas GR2 has the pre-pro*-* region from Sap2. Our data suggest that both the Hex1 and Sap2 signal sequences work in the context of these synthetic GR reporters (Figs. 2 and 3).

The GR1 and GR2 reporters carry other features that are designed to facilitate their exploitation as *N-*glycosylation reporters (Fig. 1). Convenient restriction sites have been engineered to facilitate the introduction of alternative sequences in the reporter genes (Fig. 1). The reporters carry carboxy-terminal FLAG_3_ and His_6_ tags that facilitate their detection (Fig. 2) and partial purification (Fig. 4C). Functional trypsin cleavage sites (Fig. 3C) are located just upstream and downstream of the single *N-*glycosylation site (Fig. 1), facilitating downstream analysis by mass spectroscopy. However further purification of the GR reporter proteins would be required for this type of analysis, and the following issues might be considered in the design of such experiments. Firstly, the purification might be performed at 4 °C and protease inhibitors included to reduce proteolysis. Secondly, secreted proteins should be concentrated from 4 to 5 l of culture supernatant, for example by ultra-filtration, before affinity chromatography using the His_6_ tag. Thirdly, an additional purification step, possibly based on immuno-affinity chromatography of the FLAG-tag (Einhauer and Jungbauer, 2001), might be required to remove endogenous *C. albicans* proteins that co-purify with GR reporters on the nickel columns. Even after purification, some heterogeneity in *N-*glycosylation patterns is likely to remain, because individual GR molecules probably vary in their *N*-glycan structures. Nevertheless, our system simplifies this heterogeneity by facilitating the analysis of a specific GR sequence carrying a single *N-*glycosylation site. Therefore, these GR reporters should prove to be useful tools for the dissection of *N-*glycosylation in *C. albicans* and its interactions with the host. We also note that the GR reporters may prove useful for mechanistic analyses of other virulence-related related processes in *C. albicans* such as secretion, cell wall biogenesis and adhesion.

## Figures and Tables

**Table 1:** *C. albicans* strains.

Strain	Genotype	Source
CAI4	*ura3*Δ*::λimm434/ura3*Δ*::λimm434*	Fonzi and Irwin (1993)
NGY98	*ura3*Δ*::λimm434/ura3*Δ*::λimm434, pmr1Δ::hisG/pmr1Δ::hisG*	Bates et al. (2005)
NGY112	*ura3*Δ*::λimm434/ura3*Δ*::λimm434, mnt1-mnt2Δ::hisG/mnt1-mnt2Δ::hisG*	Munro et al. (2005)
NGY205	*ura3*Δ*::λimm434/ura3*Δ*::λimm434, och1Δ::hisG/och1Δ::hisG*	Bates et al. (2006)
CAI4 − GR1	*ura3*Δ*::λimm434/ura3*Δ*::λimm434, RPS1*-pACT1-GR1	This study
CAI4 − GR2	*ura3*Δ*::λimm434/ura3*Δ*::λimm434, RPS1*-pACT1-GR2	This study
CAI4 − GR3	*ura3*Δ*::λimm434/ura3*Δ*::λimm434, RPS1*-pACT1-GR3	This study
NGY98 + GR1	*ura3*Δ*::λimm434/ura3*Δ*::λimm434, pmr1Δ::hisG/pmr1Δ::hisG, RPS1*-pACT1-GR1	This study
NGY112 + GR1	*ura3*Δ*::λimm434/ura3*Δ*::λimm434, mnt1-mnt2Δ::hisG/mnt1-mnt2Δ::hisG, RPS1*-pACT1-GR1	This study
NGY205 + GR1	*ura3*Δ*::λimm434/ura3*Δ*::λimm434, och1Δ::hisG/och1Δ::hisG, RPS1*-pACT1-GR1	This study
NGY98 + GR2	*ura3*Δ*::λimm434/ura3*Δ*::λimm434, pmr1Δ::hisG/pmr1Δ::hisG, RPS1*-pACT1-GR2	This study
NGY112 + GR2	*ura3*Δ*::λimm434/ura3*Δ*::λimm434, mnt1-mnt2Δ::hisG/mnt1-mnt2Δ::hisG,* R *RPS1*-pACT1-GR2	This study
NGY205 + GR2	*ura3*Δ*::λimm434/ura3*Δ*::λimm434, och1Δ::hisG/och1Δ::hisG, RPS1*-pACT1-GR2	This study
