# Supplementary material for: Reporters for the analysis of N-glycosylation in Candida albicans
Source: Fungal Genet Biol. 2013 Jul;56:107–15. doi: 10.1016/j.fgb.2013.03.009 (PMC3705205; doi:10.1016/j.fgb.2013.03.009)
Supplement: Supplementary data 1 [file mmc1.pdf]

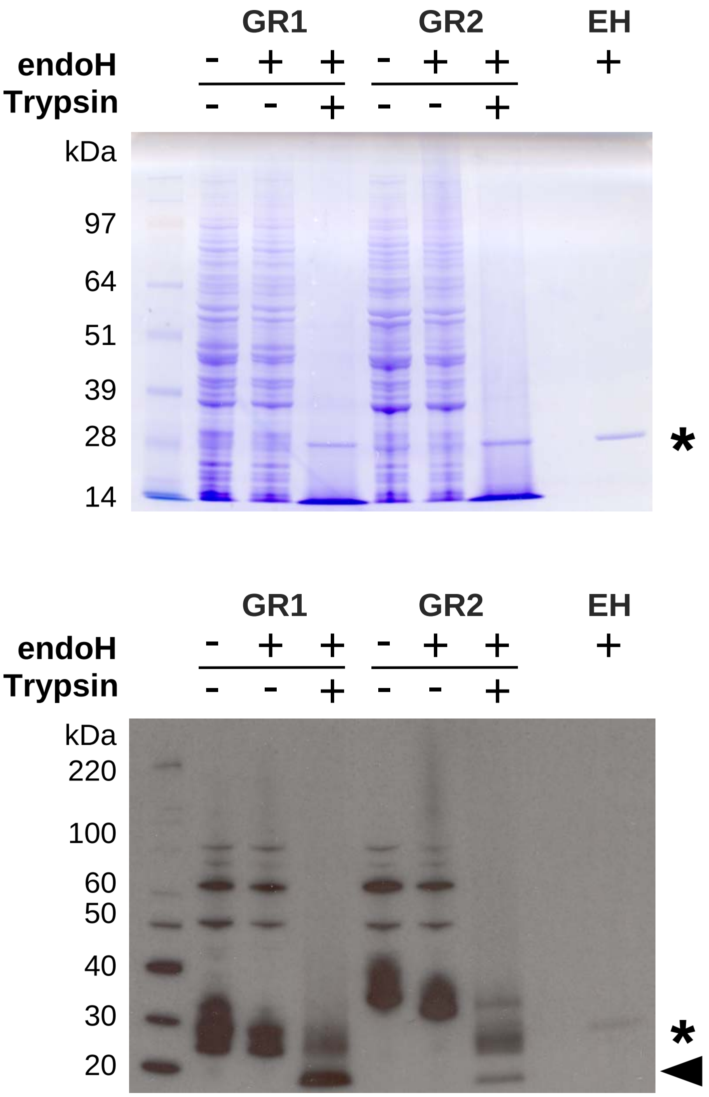

Fig. S1. Coomassie staining (upper panel) of an SDS-PAGE gel used for western blotting of trypsin digested extracts from *C. albicans* pACT-GR1 and pACT-GR2 cells with an anti-FLAG antibody (lower panel, corresponding to Fig. 2C). The carboxy-terminal FLAG-tagged GR tryptic peptides of about 15 kDa are highlighted (arrow) as well as the endoglycosidase H band at about 29 kDa (asterisk). The Coomassie stained gel confirms the efficacy of the tryptic digestion and the comparable protein loading of GR1 and GR2 extracts. Size standards for gel were pre-stained protein standards (SeeBlue® Plus2: Life Technologies, UK), and the size standards for the western Blot were MagicMark™ XP Western Protein Standard (Life Technologies, UK).

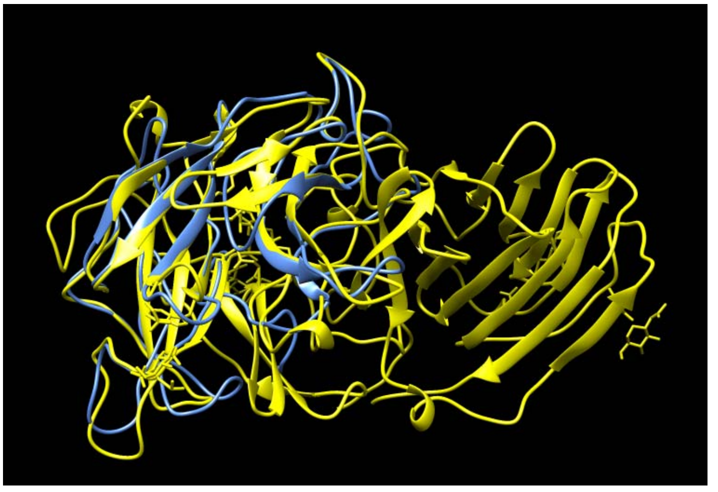

Local **RMSD**				
	Alpha Carbons	Back Bone	Heavy	All
RMSD	2.04	2.01	2.54	2.54
Atoms	74	296	488	488
Structure	Residues			
1Y4W chain 'A'	197-255, 256-270			
GR1-PDB	90-148, 150-164			

Fig. S2. Structural prediction of the aligned GR1 reporter protein with its closest match which is *Aspergillus awamori* exo-inulinase (1Y4W chain 'A'), using SuperPose software (version 1.0: Maiti R. *et al.*, 2004), yielding the indices presented in the table. The Local RMSD (Root Mean Square Deviation: Zhang Y *et al.* 2005) represents the comparison between the GR1 protein and the homologous region of *A. awamori* exo-inulinase. The unaligned domain of this exo-inulinase (to the right in the figure) is not included in this structural comparison.

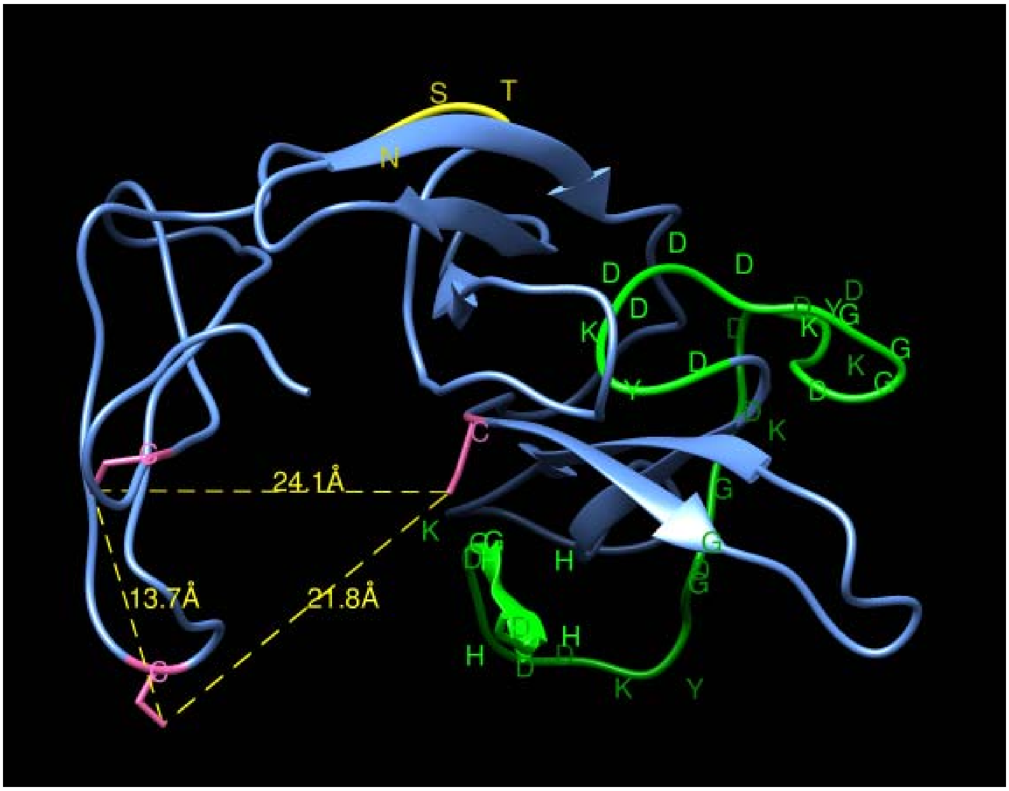

Fig. S3. Structural prediction of the GR1 reporter protein lacking the signal sequence, highlighting the distance between the cysteine residues within this predicted structure (13.7, 21.8 and 24.1 Å, respectively).
